# Optimal Fluorescence Waveband Determination for Detecting Defective Cherry Tomatoes Using a Fluorescence Excitation-Emission Matrix

**DOI:** 10.3390/s141121483

**Published:** 2014-11-14

**Authors:** In-Suck Baek, Moon S. Kim, Hoosoo Lee, Wang-Hee Lee, Byoung-Kwan Cho

**Affiliations:** 1 Department of Biosystems Machinery Engineering, College of Agricultural and Life Science, Chungnam National University, 99 Daehak-ro, Yuseong-gu, Daejeon 305-764, Korea; E-Mails: huntersp@naver.com (I.-S.B.); benet33@naver.com (H.L); wanghee@cnu.ac.kr (W.-H.L.); 2 Environmental Microbiology and Food Safety Laboratory, Agricultural Research Service, U.S. Department of Agriculture, Powder Mill Road, Building #303, BARC-East, Beltsville, MD 20705, USA; E-Mail: moon.kim@ars.usda.gov

**Keywords:** cherry tomato, quality sorting, defect detection, hyperspectral image, fluorescence image

## Abstract

A multi-spectral fluorescence imaging technique was used to detect defective cherry tomatoes. The fluorescence excitation and emission matrix was used to measure for defects, sound surface and stem areas to determine the optimal fluorescence excitation and emission wavelengths for discrimination. Two-way ANOVA revealed the optimal excitation wavelength for detecting defect areas was 410 nm. Principal component analysis (PCA) was applied to the fluorescence emission spectra of all regions at 410 nm excitation to determine the emission wavelengths for defect detection. The major emission wavelengths were 688 nm and 506 nm for the detection. Fluorescence images combined with the determined emission wavebands demonstrated the feasibility of detecting defective cherry tomatoes with >98% accuracy. Multi-spectral fluorescence imaging has potential utility in non-destructive quality sorting of cherry tomatoes.

## 1. Introduction

International agricultural quality competitions have become a popular way to increase free trade between nations. Consumption and exports of cherry tomatoes are increasing as consumers become more health-conscious and seek convenient healthy foods. Cherry tomatoes produced in Korea are largely exported to other Asian countries. Quality and safety are important concerns for produce exports. Selecting quality produce and careful shipping are critical to maintaining a competitive edge in export countries. There are far fewer methods for quality selection in cherry tomatoes than there are in standard tomatoes.

Cherry tomato quality is measured by peel coloration, shape, size, cracking, and sugar/acid content; however, current selection techniques typically involve simple size and shape discrimination with a drum selector. There is no more comprehensive technology for measuring quality in this product. Cracked tomatoes swiftly reduce quality when they are not detected prior to distribution, because infections lodged in the cracks are spread throughout the batch, reducing unit sales. Thus, cracked tomatoes must be selected and removed prior to shipment. Selection of cherry tomatoes is problematic due to their small size and large quantity, which prohibits the use of selection technologies used in standard tomatoes. Current nondestructive selection technologies employ spectroscopy and mechanical visualization, although these methods are difficult to apply at the site of production. Spectroscopy requires frequent recalibration and repeated sample measurements, and only measures a fixed point, rather than the entire surface of the fruit [1]. Mechanical visualization can be used to observe the entire fruit surface, but can provide only basic surface information. In cracked tomatoes, the surface and interior colors are similar, making general color imaging difficult. Thus, a new nondestructive selection technology is needed.

Hyperspectral image is a novel technology that collects image data at a series of narrow and contiguous wavelength bands for each spatial pixel of a captured scene, constructing 3-dimensional hyperspectral cube, to acquire both spatial and spectral information simultaneously [2]. As the spectrum of each spatial pixel can characterize the target substances at corresponding spot on the hyperspectral image, this technology can be used to identify and detect spectral and spatial anomalies in agricultural products [2]. Hyperspectral imaging can provide physical and chemical information beyond that provided by the simple optical R/G/B regions used by standard color cameras. It is even possible for the hyperspectral fluorescence characteristics of an object to yield a detailed image [3]. Hyperspectral fluorescence imaging technology combines fluorescence and digital imaging to provide simultaneous physical and chemical data. The technology is used for various applications, including diagnostics, remote imaging, and agricultural quality and safety monitoring. Agricultural applications of multispectral imaging include detection of apples infected with stock stool [4], detection of internal organ residue on the surface of chicken carcasses [5,6], and assessment of pickle quality [7].

The light source is very important in hyperspectral fluorescence techniques, because powerful, narrowband excitation is required to yield sufficient light emission from the sample [8]. Emission intensity is greatly influenced by excitation power and exposure time. Thus, the optimal excitation wavelength and a powerful light source must be identified in the context of the fluorescence characteristics of the sample type.

We validated the optimal excitation and emission wavelengths for detection of cracked cherry tomatoes by fluorescence imaging. The specific objectives were: (1) to measure the fluorescence characteristics of cherry tomato (*i.e.*, sound surface, stems, cracks) at excitation wavelengths between 200 nm and 675 nm and emission wavelengths between 225 nm and 700 nm; (2) to use a hyperspectral fluorescence imaging system equipped with an optimal light source for detection; and (3) to establish appropriate image processing and detection algorithms for defective cherry tomatoes.

## 2. Materials and Methods

### 2.1. Sample Preparation

‘Koko’ cherry tomatoes were harvested from a farm in Buyeo, Chungnam, South Korea. Cherry tomatoes were placed in plastic bags with ice and transported to the laboratory. The harvests were divided into groups. The first group contained 16 naturally cracked cherry tomatoes by random sampling and was used to identify the fluorescence characteristics of each part of the tomato. The second group consisted of 121 cracked and 23 normal cherry tomatoes, used to validate the selection accuracy of multispectral fluorescence imaging. Cuticle defects were arranged in an upward-facing position for ideal imaging.

### 2.2. Measurement of Fluorescence Spectra

**Figure 1.**
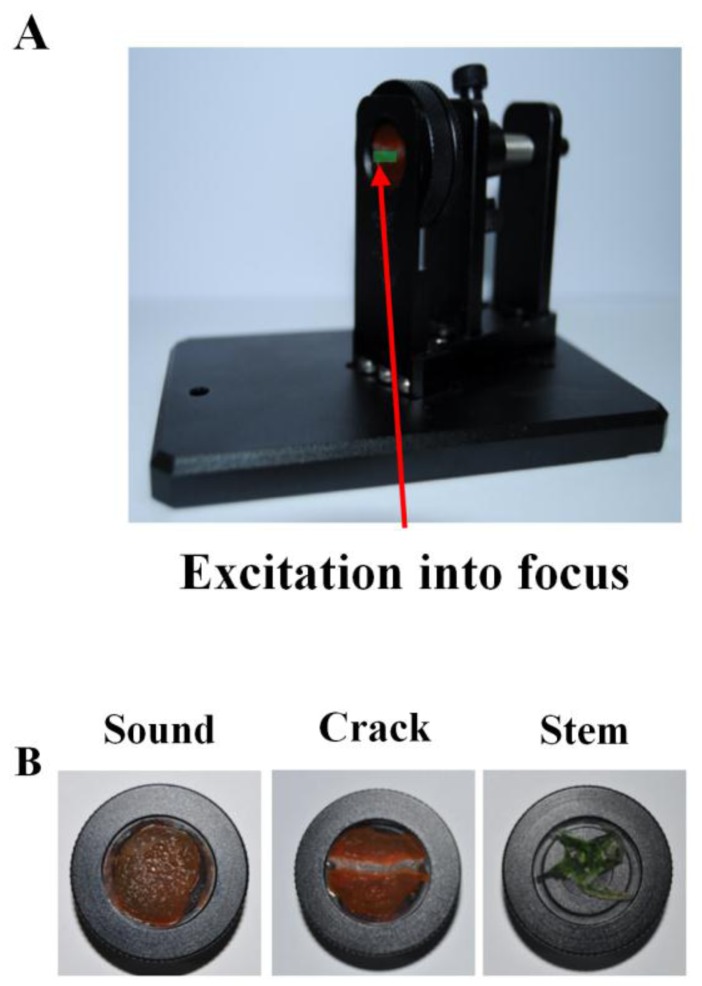
Photos of cell unit (**A**); precision cell with samples (**B**).

Normal cuticle and stem parts and released matrix, fluoroMate FS-2 (Scinco Co. Seoul, Korea) were used to measure cuticle defects. To measure the fluorescence characteristics of each part of the fruit, samples were thinly cut and measured after placing the samples in circular cells made of a non-fluorescent substance as in Figure 1. A fluorophotometer measures fluorescence by measuring emitted photons at the detection wavelength. In this study, the fluorometer photo multiplier tube (PMT) was 700 volts and integration time was 20 msec. Excitation wavelengths from 200 nm to 665 nm were emitted by 150 W continuous xenon lamp and 5 nm intervals; emitted light from the sample was measured in 1 nm intervals from 225 nm to 700 nm. Relative Fluorescence Intensity (RFI) measured across these wavelengths was used to build an Excitation-Emission matrix (EEM).

### 2.3. Measurement of Fluorescence Images

**Figure 2.**
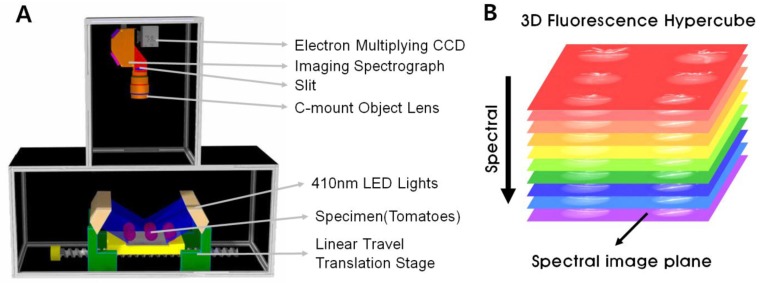
Schematic of the hyperspectral imaging system (**A**) and a structure of the 3D Hypercube (**B**).

The hyperspectral imaging system was used to acquire fluorescence images as shown in Figure 2. The system was designed for use at 450 nm to 700 nm [9]. The system includes an electron multiplying charge-coupled device camera (EMCCD: Luca R DL-604M, 14-bit, Andor Technology, South Windsor, CT, USA), a C-mount objective lens (F1.9 35-mm compact lens, Schneider Optics, Hauppauge, NY, USA), imaging spectrograph (VNIR, Headwall photonics, Fitchburg, MA, USA), programmable uniaxial stage, and light sources (Figure 2A). Emission intensity is determined by the power of the light source and exposure time; thus, a real-time detection system must have a proper light source and short exposure time. To obtain sufficient excitation for imaging, we used five 410 nm 10 W LEDs (LZ4-40UA10, Ledengin Inc., Mansfield, OH, USA). As in Figure 2A, a pair of light sources was used. Wavelengths over 430 nm and the long wavelength spectral tail from the LEDs were blocked by a low-pass filter (<445 nm, 70% transmission with 450 nm cut-off) in front of the LEDs. Only spectral signals up to 700 nm were acquired, eliminating second-order excitation effects. The dimension of the light source was 250 mm (Length) × 100 mm (Width) × 150 mm (Height). Selecting the wavelength of LED excitation was performed by fluorophotometry (details provided in the Results and Discussion section).

Visual Basic 6.0 was used to run the hyperspectral imaging system. To set the exposure time for hyperspectral imaging, the step motor was employed by automatically calculating the motion and exposure times. The image was projected through a C-mount objective lens and high-pass 460 nm filter (>455 nm, 70% transmission with 460 nm cut-off), a slit, and imaging spectrograph. The instantaneous field of view (IFOV) of the system was limited to a thin line through the spectrograph aperture slit (60 μm × 18 mm) between the C-mount objective lens. Imaging then comprised each spatial location on the IFOV of a line-scan and spectral information at each wavelength was determined by the spectrograph. The two-dimensional spectral and spatial data on the IFOV were captured by the EMCCD and stored as an image in the computer. The collected two-dimensional data was used to generate a 3D fluorescence hypercube (Figure 2B).

Spectral calibration was performed using a general-purpose cool-white fluorescent lamp with emitted wavelengths for terbium (Tb^3+^), europium (Eu^3+^), and mercury (Hg). The white Teflon panel emitted a peak wavelength after illumination with the cool-white fluorescent lamp and numbers in the spectral dimension of the hyperspectral system corresponded to actual wavelength peaks from the cool-white fluorescence lamp after linear regression.

In this study, the exposure time was 0.1 s and the motor moved at 1 mm intervals for 250 steps. We obtained a full hyperspectral image of 25 cm (whole sample). Images provided mass information, known as a 3D fluorescence hypercube. The hypercube provides 2D fluorescence imaging (from 470 nm to 700 nm) and spectral information (Figure 2B). The optimum wavelength range for defect tomato detection was determined from the fluorescence investigation and release matrix.

### 2.4. Data Analysis

EEM data was stored as Unsigned 64-bit integers; the 14-bit hyperspectral image was stored and analyzed as Unsigned 16-bit integers. The EEM and hyperspectral image data were processed in Matlab software (version 7.0.4, Mathworks, Natick, MA, USA). The optimum excitation waveband was selected by analyzing the EEM data by two-way ANOVA and principal component analysis (PCA). In this study, EEM data analysis yielded total emission characteristics according to the excitation of each part of the fruit (e.g., crack *vs.* sound, crack *vs.* stem). We then analyzed and selected an excitation waveband that could distinguish defect tomatoes by fluorescence solidity values of parts (sound surface, stem, defect area). To select the excitation waveband in the hyperspectral image system, we extracted spectral information from each part (sound surface, stem, crack area) in the hyperspectral fluorescence image. Data was analyzed by the PCA method to select the optimal emission waveband for detection of cherry tomato surface defects [10].

## 3. Results and Discussion

### 3.1. Fluorescence Attributes of Stem, Sound Skin, and Cracks

**Figure 3.**
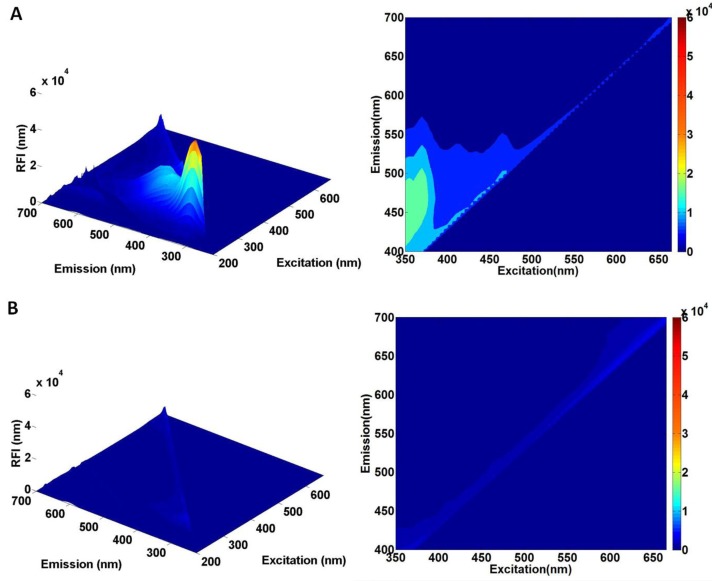

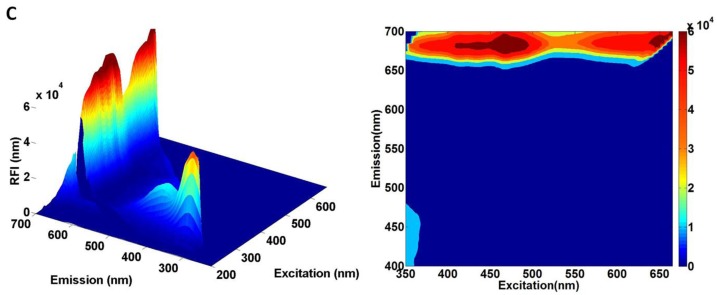
Averaged EEM of each data set of (**A**) crack; (**B**) sound skin; (**C**) stem with the spectral rage of excitation from 200 to 655 nm and emission from 225 to 700 nm.

Three-dimensional (3-D) graphs for the average fluorescence emission and excitation matrix of defect tomato, stem, and normal cuticle were plotted with respect to excitation wavelength (x-axis), emission wavelength (y-axis), and RFI (z-axis) (Figure 3). The right contour plot in Figure 3 indicates the excitation (350∼665 nm) and emission wavelengths (400∼700 nm) used in this study. At excitation 250∼300 nm, defect and stem parts release UV fluorescence (300∼400 nm), similar to the reaction of general proteins [11]. However, the protein is not target component to detect crack, we did not used excitation below 350 nm. Defect parts could be detected by fluorescence at 350∼580 nm (with excitation 350∼480 nm) due to the flavonoids, carotenoids, phenolic compounds, cell walls, and anthocyanins [12]. In detail, flavonoid emission band were identifiable at 300, 450∼500 and 570∼580 nm with excitation at 266, 355, and 480 nm, respectively, while anthocyanin fluorescent spectrum was peaked at 380 and 450 at 266 and 355–400 nm of excitations, respectively. The carotenoids spectrum was observed at the range from 420 to 580 nm. Also, cell walls were responsible for fluorescence emission at 450∼500 nm [13]. In addition, the spectral patterns and intensity of the compounds are different. As the objective of this study is to distinguish defect from other areas, the spectrum of these compounds do not have to be differentiated. Rather, it is important that the spectrum of them allow us to detect defect areas from stem and sound surface. Stems emitted 650∼700 nm of fluorescence at excitation wavelengths ranged from 350 nm to 650 nm because of the presence of chlorophyll [14]. However, emission from intact cuticle is too weak to detect fluorescence. This might be due to waxy nature of intact cuticle as previously reported that the light and UV absorbance of waxes were drastically reduced and reached almost zero above about 350 nm [15].

### 3.2. Selection of the Excitation Waveband

**Figure 4.**
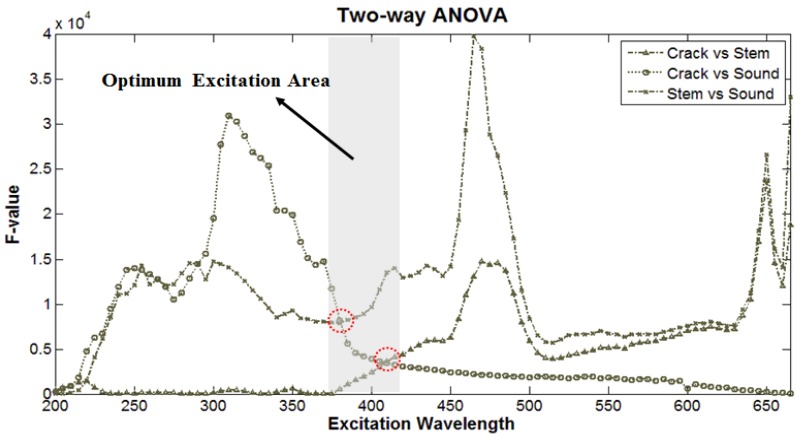
F values of wavelength used for discriminating defect area from sound and stem on the cherry tomatoes.

The optimal excitation and emission wavelengths have been selected to efficiently distinguish defect, stem, and normal cuticle. The emission and excitation of each part of the cherry tomato was characterized by F-value obtained from ANOVA with comparing two groups (Figure 4). A large F value indicates a more statistically significant mean separation between groups [16]. To eliminate intact parts and distinguish them from cracked parts in fluorescence images, we compared crack with stem and sound surface using ANOVA. The crack and stem were distinguishable at 480 nm excitation because of the presence of chlorophyll detectable by red to far-red fluorescence. Cracks and sound surfaces were clearly distinguishable at 380 nm excitation. This is because that fluorescence emission from cracked parts exists in the blue region while there is no fluorescence from a sound surface with the excitation wavelength of 380 nm.

In order to find optimal excitation waveband, two-way ANOVA method between the two groups (e.g., crack area *vs.* stem) was used. Emission characteristics by excitation waveband of each part were expressed as F-values in pairwise group comparisons (Figure 4). The highest F-value for discriminating defect areas from stem areas was 320 nm, and the F-value for distinguishing stem from sound surface and crack was peaked at 460 nm. Consequently, the optimal excitation wavebands for differentiating 3 areas of tomatoes (defect, stem and sound areas) were 380∼410 nm determined at the intersection points of two plots in Figure 4.

### 3.3. Analysis of Fluorescence Images

**Figure 5.**
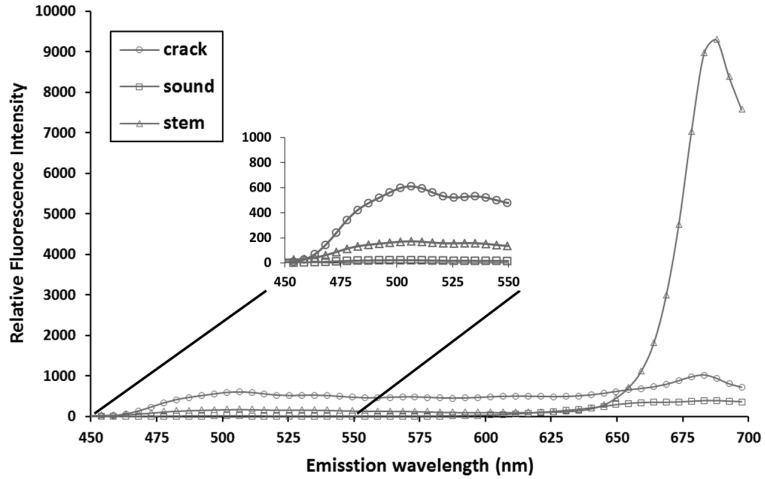
Mean fluorescence emission spectra of sound surface, defect area, stem area in hyperspectral image of cherry tomatoes upon excitation by 410 nm light source.

Figure 5 illustrates the average fluorescence spectrum of each part (defect area, sound surface, and stem) extracted from hyperspectral fluorescence images obtained with 410 nm excitation wavelength. In the blue-green spectrum (480∼550 nm), defect tomato parts yielded strong fluorescence, while stem emitted strong fluorescence in the red region (680∼700 nm) (Figure 5). However, fluorescence of the intact fruit was consistently low. The results showed similar patterns with the results derived from EEM analysis.

**Figure 6.**
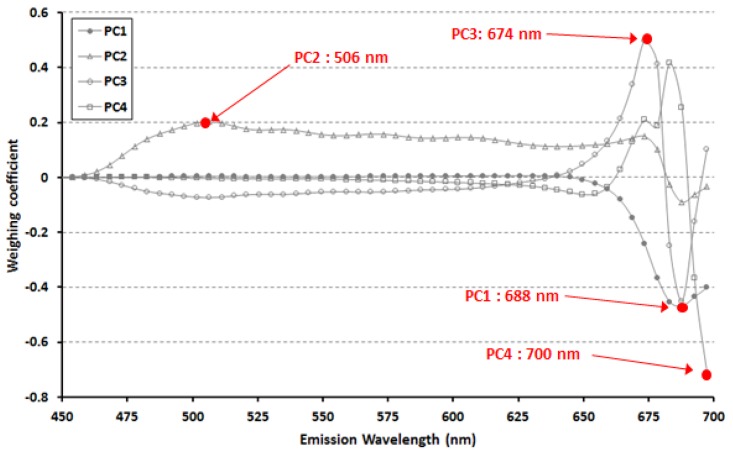
Spectral weighting coefficients for principal components.

To detect defect tomatoes using the emission spectrum at 410 nm excitation, we selected the significant multi-waveband analyzed by PCA. Figure 6 shows the weighting coefficients for PCA (PC1–PC4). The peaks and valleys signify the dominant waveband for each PC which transforms the original wavelengths by a linear sum at each wavelength multiplied by calculated weighting coefficients. The maxima for each PC were observed at 688 nm for PC1, 506 nm for PC2, 674 nm for PC3, and 700 nm for PC4. Fluorescence release was the greatest from stem with the maximum weighting coefficient of PC1 at 688 nm, and the weighting coefficient of PC2 appeared at 506 nm, the fluorescence wavelength of cracked tissue. These results were consistent with the EEM analysis.

### 3.4. Detection of Defect Cherry Tomatoes

**Figure 7.**
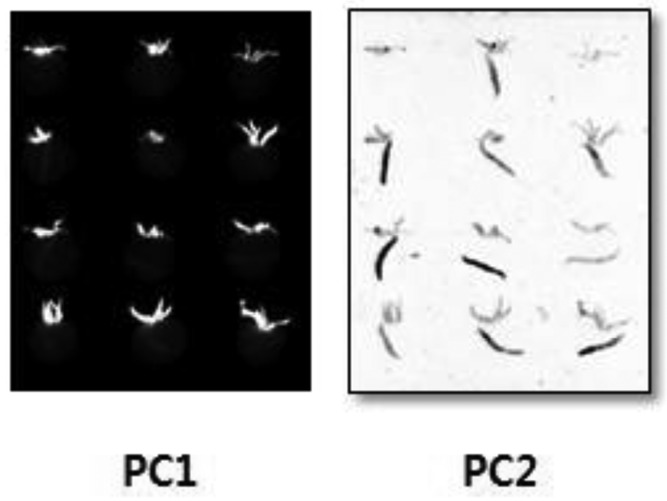
PC images by using two wavebands.

**Figure 8.**
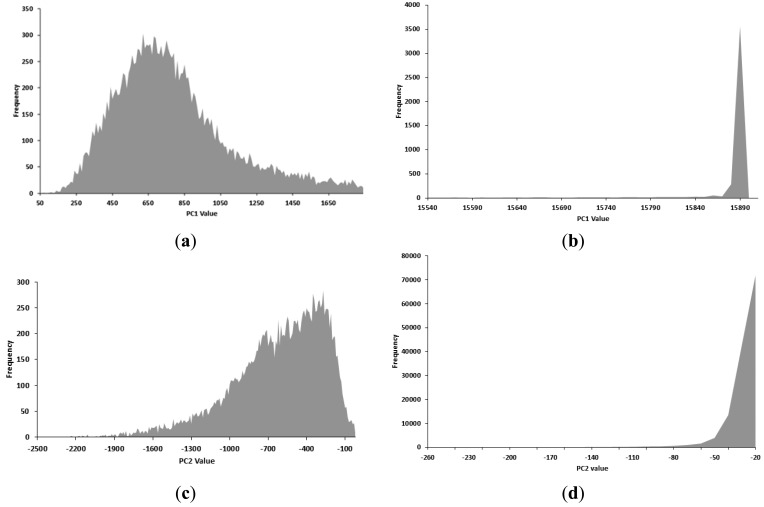
PC value about each part by histogram: (**a**) crack (PC1); (**b**) stem (PC1); (**c**) crack (PC2); and (**d**) sound area (PC2). The x-axis indicates PC value and the y-axis indicates frequency.

Detection of damaged areas is difficult with a single spectral band. Hence, PCA was performed for the entire spectral data of defect, stem and sound surface. A principal component (PC) image was generated from the data at 688 nm and 506 nm (Figure 7). As aforementioned, PC is a new variable created by linear combination of original variables, *i.e.*, emission wavelengths. Different numbers in PC means they are generated by different coefficient in linear combination. Generally, higher PC can explain more variations in data. The total image for PC1 yielded a precise image of the stem, and defects and stems appeared in the PC2 image. To detect defect tomatoes, the PC2 image after erasing stem part using the PC1 image was used and a histogram was produced by extracting the PC1 and PC2 values for stems, cracks, and sound surface (Figure 8). The PC1 value of cracks was distributed across 50–1700, while the PC1 value of stems was concentrated around 15,800. In the PC2 image, the value for cracks was distributed from −1900 to −100, and the value for sound surfaces peaks from −50 to −20.

**Figure 9.**
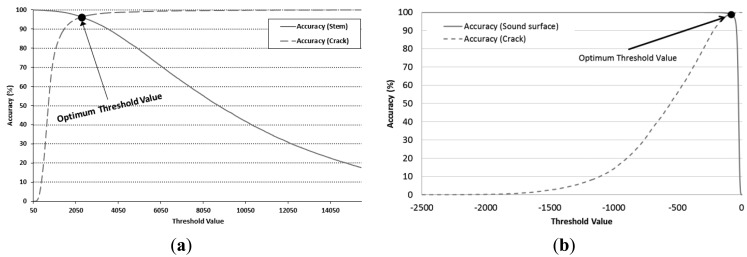
Classification aaccuracy when threshold value of two groups is applied: (**a**) stem *vs.* crack; (**b**) sound area *vs.* crack.

To generate a binary image by applying threshold value of each PC image, it was necessary to establish a global threshold value that could distinguish the two groups. We expressed the distinction accuracy with the applied threshold in Figure 9; the optimal thresholds were 2175 and −113 (points of intersection) for discriminating stem from crack areas and sound surfaces from crack areas, respectively.

**Figure 10.**
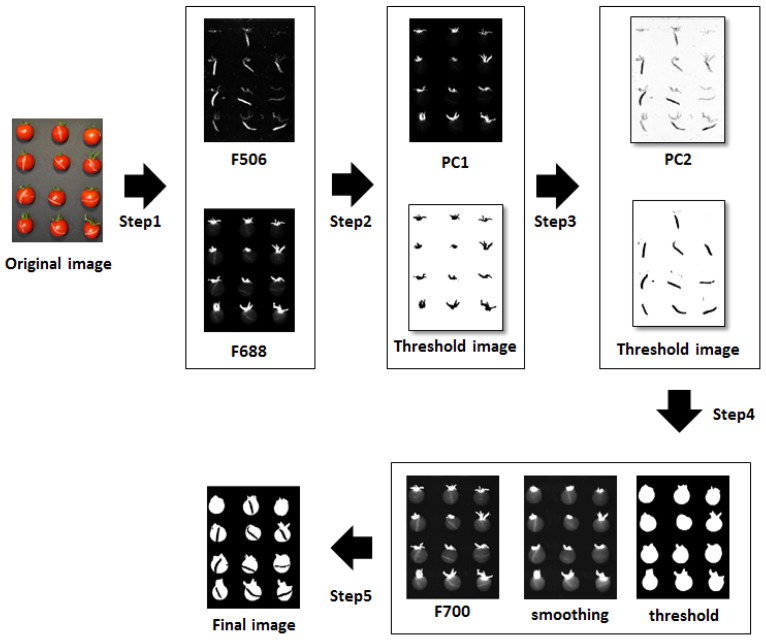
Image processing for detecting defects of cherry tomatoes.

Defect areas on the cherry tomatoes were detected by using multispectral image processing methods (Figure 10). The experimentally derived fluorescence images at 506 and 688 nm were used to generate PC images so that the stem and the crack areas were emphasized. The PC1 and PC2 binary images were generated by applying the global threshold values to detect the stem and the crack areas only. The PC1 binary image was used to erase the stem parts from the PC2 image so that only the crack areas could be detected. In addition, waveband images of 700 nm were subjected to a smoothing method, followed by thresholding at a specific threshold value to discriminate samples from background. The threshold image was used to apply a mask for the resultant PC2 binary image. The masked PC2 binary image was the resultant image displaying only the defect areas on the cherry tomatoes.

**Figure 11.**
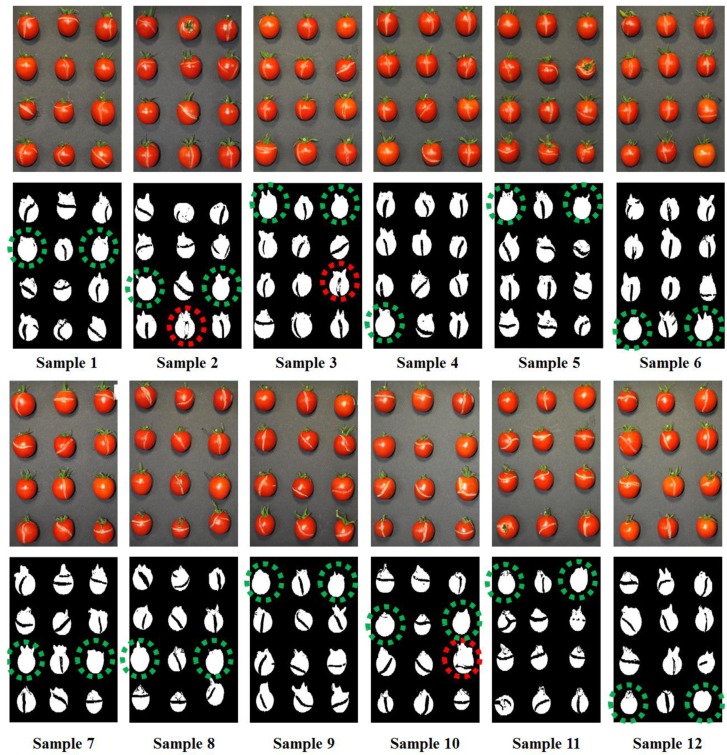
Resultant detection images.

The actual sample images and detection results are displayed in Figure 11. The green circle in the binary image indicates a normal cherry tomato with no defects. In contrast, red circles indicate defect tomatoes. With this process, 121 defect and 23 normal tomatoes were imaged, resulting that defect tomatoes could be detected with >98% accuracy (Table 1). Three failures might be due to subtle fluorescence differences between defects and sound surface, resulting in the detection error. To improve the accuracy, even though it's still enough high, the hyperspectral fluorescence imaging system or statistical algorithm might be adjusted.

## 4. Conclusions

The goal of this study was to develop a simple image processing method based on multispectral fluorescence imaging for onsite detection of cuticle defects in cherry tomatoes. We found that cherry tomato features yield significant fluorescence emission wavebands at specific excitation wavelengths. These attributes enable discrimination of defect from intact cherry tomato surfaces. Excitation at 410 nm was used to distinguish defects from the sound surfaces and stems. To detect defects on cherry tomatoes by using multispectral fluorescence images, the optimal wavebands were determined by PCA to be 688 and 506 nm. Dual-fluorescence imaging at 688 and 506 nm provided defect detection with 98% accuracy. We have demonstrated the feasibility of using hyper- and multispectral imaging to detect defect cherry tomatoes. The technology can be applied for quality control in cherry tomato production.

## Figures and Tables

**Table 1.** Classification results.

**Intact Cherry Tomatoes**	**Defect Cherry Tomatoes**	**Accuracy (%)**
23/23	118/121	98
